# A first-in-human clinical study of laparoscopic autologous myoblast sheet transplantation to prevent delayed perforation after duodenal endoscopic mucosal dissection

**DOI:** 10.1186/s13287-024-03730-3

**Published:** 2024-04-23

**Authors:** Kengo Kanetaka, Yasuhiro Maruya, Miki Higashi, Shun Yamaguchi, Ryo Matsumoto, Shinichiro Kobayashi, Keiichi Hashiguchi, Fumiya Oohashi, Masaki Matsumura, Takahiro Naka, Yusuke Sakai, Kazuhiko Nakao, Shigeru Miyagawa, Susumu Eguchi

**Affiliations:** 1 Tissue Engineering and Regenerative Therapeutics in Gastrointestinal Surgery, Nagasaki University Graduate School of Biomedical Sciences, Sakamoto 1-7-1, 8528102 Nagasaki, Japan; 2 Department of Surgery, Nagasaki University Graduate School of Biomedical Sciences, Nagasaki, Japan; 3 Department of Gastroenterology and Hepatology, Nagasaki University Graduate School of Biomedical Sciences, Nagasaki, Japan; 4 Terumo Corporation, Shibuya, Japan; 5 Department of Chemical Engineering, Faculty of Engineering, Graduate School, Kyushu University, Fukuoka, Japan; 6 Department of Cardiovascular Surgery, Osaka University Graduate School of Medicine, Osaka, Japan

**Keywords:** Endoscopic submucosal resection, Superficial non-ampullary duodenal epithelial tumor, Laparoscopy, Cell-sheet transplantation, Clinical trial

## Abstract

**Background:**

The detection rate of superficial non-ampullary duodenal epithelial tumors (SNADETs) has recently been increasing. Large tumors may contain malignant lesions and early therapeutic intervention is recommended. Endoscopic mucosal dissection (ESD) is considered a feasible treatment modality, however, the anatomical and physiological characteristics of the duodenum create a risk of postoperative perforation after ESD.

**Methods:**

To explore whether myoblast sheet transplantation could prevent delayed perforation after ESD, a first-in-human (FIH) clinical trial of laparoscopic autologous myoblast sheet transplantation after duodenal ESD was launched. Autologous myoblast sheets fabricated from muscle tissue obtained seven weeks before ESD were transplanted laparoscopically onto the serous side of the ESD. The primary endpoints were the onset of peritonitis due to delayed perforation within three days after surgery and all adverse events during the follow-up period.

**Results:**

Three patients with SNADETs ≥ 20 mm in size underwent transplantation of a myoblast sheet onto the serous side of the duodenum after ESD. In case 1, The patient’s postoperative course was uneventful. Endoscopy and abdominal computed tomography revealed no signs of delayed perforation. Despite incomplete mucosal closure in case 2, and multiple micro perforations during ESD in case 3, cell sheet transplantation could prevent the postoperative massive perforation after ESD, and endoscopy on day 49 after transplantation revealed no stenosis.

**Conclusions:**

This clinical trial showed the safety, efficacy, and procedural operability of this novel regenerative medicine approach involving transplanting an autologous myoblast sheet laparoscopically onto the serosa after ESD in cases with a high risk of delayed perforation. This result indicates the potential application of cell sheet medicine in treating various abdominal organs and conditions with minimal invasiveness in the future.

**Trial registration:**

jRCT, jRCT2073210094. Registered November 8 2021,

https://jrct.niph.go.jp/latest-detail/jRCT2073210094.

## Introduction

Owing to its rarity, the duodenum has not received much attention as a site of tumor development. The incidence of superficial non-ampullary duodenal epithelial tumors (SNADETs), in particular, is said to be 0.02-0.5% among autopsy series [1]. However, the rate of detection of these tumors has recently been increasing owing to advances in endoscopic technology and increasing awareness of this disease [2]. Duodenal cancer is estimated to account for 0.5% of all gastrointestinal cancers. A recent Japanese study using a large-scale national database indicated that the incidence of duodenal cancer registered in 2016 was 23.7 per 1,000,000 person-years. The authors described the incidence as increasing worldwide [3–5].

Accumulating evidence indicates that even if a tumor is localized to the mucosal layer, large tumors may contain malignant lesions [6], so early therapeutic intervention is recommended for these tumors [4]. The duodenum is located near vital organs, such as the pancreas and bile duct, and invasive procedures, such as Whipple operation, are needed to treat cases of duodenal cancer. In Japan, due to widespread esophagogastroduodenoscopy being performed to screen for gastric cancer, more than 50% of duodenal cancer cases are diagnosed at the localized stage, and endoscopic resection of these superficial tumors is recommended. According to an analysis of a national cancer registry conducted by Yoshida et al., approximately 48% of these cancers were treated by endoscopic resection, with favorable short- and long-term outcomes achieved [3].

Endoscopic resection, such as endoscopic mucosal resection (EMR) and endoscopic submucosal dissection (ESD), are considered feasible treatment modalities for these tumors. In contrast to the widespread application of ESD for early gastric and colorectal cancer, ESD for duodenal tumors has been hampered by the fact that maneuvering the scope is difficult because of the narrow curling lumen and very thin wall of the duodenum. Furthermore, mucosal defects after ESD are exposed to irritant digestive contents, such as bile and pancreatic juices, which impair the integrity of the remnant duodenal wall. These anatomical and physiological characteristics create a risk of postoperative perforation after ESD, which is reported in 0-14% of cases [7] and if delayed perforation developed emergency surgery is often required to cure lethal peritonitis [8–10]. Clip closure or coverage with artificial materials for post-ESD ulcers has been attempted in order to prevent perforation [4], with favorable outcomes of a decreased incidence of delayed complications achieved [11]. However, Kato et al. reported that mucosal closure may be difficult in some tumors, such as those located in the proximal portion or those with large, occupied circumferences [12].

Mizutani et al. analyzed the risk factors for incomplete closure and found that a lesion location at the medial/anterior wall and a large lesion size were independent predictors of incomplete closure [13]. Shielding the wound with a Polyglycolic acid (PGA) sheet is also reportedly effective in avoiding delayed adverse events when complete closure of the mucosal defect is not possible. However, Fukuhara reported that the short-term outcomes of these patients were worse than those of patients who achieved complete closure [14].

Laparoscopic endoscopy cooperative surgery (LECS) is an alternative treatment approach that can achieve an appropriate resection margin and prevent duodenal leakage by reinforcing the ESD site. Kanaji et al. reported the safety and feasibility of duodenal LECS in a single-center prospective study [15], and a retrospective multicenter study confirmed its safety and feasibility [16]. However, closure of the thin wall after duodenal ESD remains challenging and requires highly advanced laparoscopic suturing techniques. Furthermore, a large mucosal defect encompassing the posterior wall or the pancreatic side can be difficult to suture, and great care should be taken to avoid causing stenosis or involving the papilla.

Although the precise mechanism underlying delayed perforation is unclear, irritating substances, such as bile and pancreatic juice, may play a crucial role [14, 17, 18]. In addition, Hanaoka et al. reported that delayed perforation after ESD occurred because of ischemic changes at the ESD site [19]. In a large animal model, we revealed that ischemia is one of the main causes of delayed perforation after duodenal ESD [20].

We previously reported the efficacy of myoblast sheet transplantation in preventing gastric perforation and pancreatic fistula in a rat model [21, 22]. In addition, we demonstrated that transplantation of autologous myoblast sheets onto the serosal site after duodenal ESD prevented delayed perforation in a porcine model [23]. This result encouraged us to conduct a clinical trial using autologous myoblast sheets in patients with SNADETs.

We therefore established this first-in-human (FIH) clinical trial, the objective of which was to evaluate the safety, efficacy, and procedural operability of this novel regenerative medicine approach involving transplanting an autologous myoblast sheet laparoscopically onto the serosa after ESD in cases with a high risk of delayed perforation.

## Methods

### Study design

This study was a phase 1 FIH clinical trial to assess the safety and efficacy of laparoscopic myoblast sheet transplantation after duodenal ESD. This study was conducted at a single center (Nagasaki University Hospital). No sample size calculation was used in this study, but the study included six adult patients with SNADETs. This study was supported by the Japan Agency for Medical Research and Development (AMED): the title of the approved study “An exploratory clinical trial of TERGS0001 in laparoscopic and endoscopic cooperative surgery for superficial non-ampullary duodenal epithelial tumor “, registered with the Japan Registry of Clinical Trials as jRCT2073210094, and approved by the Institutional Review Board of Nagasaki University on January 20, 2021.

This study was conducted according to the principles of the Declaration of Helsinki and the Japan Good Clinical Practice guidelines, and in compliance with the ethical guidelines for medical studies in human subjects. Written informed consent was obtained from all patients.

The inclusion and exclusion criteria are listed in Table 1. Patients ≥ 20 years old with SNADETs eligible for ESD were included in this study.

**Table 1:** Inclusion and exclusion criteria of the study

Inclusion criteria+A2:C14	Exclusion criteria
1) Patients with SNADET without extension to the submucosa eligible for ESD by consensus diagnosis among specialists in gastroenterology	1) Patients who are diagnosed as SNADET on the duodenal bulbus
2) Patients with tumor size of at least 15 mm	2) Patients with muscular degenerative disease
3) Patients aged between 20 years and 80 years at the time of consent	3) Patients with metastases to lymph nodes
4) Patients who provide written informed consent for participation in this clinical trial	4) Patients with history of upper abdominal surgery
	5) Patients with hypersensitivity to Factor XIII with fibrinogen (Fibrin glue), gentamicin, amphotericin B, or human serum albumin, or with previous bovine allergy or severe metal allergy
	6) Patients undergoing treatment with coagulation accelerator (snake venom), antifibrinolytic agent, or aprotinin
	7) Patients with preexisting alcohol poisoning or drug dependence within 6 months before the provisional registration
	8) Patients with malignancy other than SNADET within 5 years before the provisional registration
	9) Patients positive for HIV-1 or HIV-22, HBV, HCV, or HTLV, patients with active infection, patients with or suspected of being infected with transmissible spongiform encephalopathy, and patients with major neurocognitive disorder
	10) Patients who are pregnant or at risk of becoming pregnant
	11) Patients with psychosis or psychiatric symptoms as complications and who are judged difficult to participate in the clinical trial
	12) Other patients who are deemed inadequate to participate in the clinical trial at the discretion of the investigator

### Outcome measurements

These assessments are presented in Table 2. Abdominal computed tomography (CT) was performed at baseline and three days after transplantation. At baseline, the tumor size was measured using an endoscopic scale. On days 1 and 7 after transplantation, an endoscopic examination was performed to assess the size of the ulcer.

**Table 2:** Treatment schedule and outcome measures

=A2:I15	Screening	Days						
		-49	0	1	3	7	At discharge	49
Informed concent	〇							
Baseline characterization	〇							
Eligibility check	〇							
Vital signs*	〇	〇	〇	〇	〇	〇	〇	〇
Blood tests†	〇	〇	〇	〇	〇	〇	〇	〇
AbdominalCT	〇				〇		〇	
Endoscopy	〇			〇		〇	〇	〇
Drain examination‡				〇	〇			
Muscle collection		〇						
Transplantation			〇					
Primary endopoint					〇			
Adverse events		→	→	→	→	→	→	→

*Blood pressure, Pulse rate, Body temperature

†WBC, RBC, Hg, Ht, PLT, AST, ALT, T-Bil, D-Bil, Amy, LDH, ALP, BUN, Cre, TG, Alb, CK, Na, K, Cl, CRP, Glu

‡Amy, T-Bil

CT: computed tomography

### Primary endpoints

- Efficacy outcome measures.

1. The onset of peritonitis due to delayed perforation within three days after surgery.

**Fig. 1:**
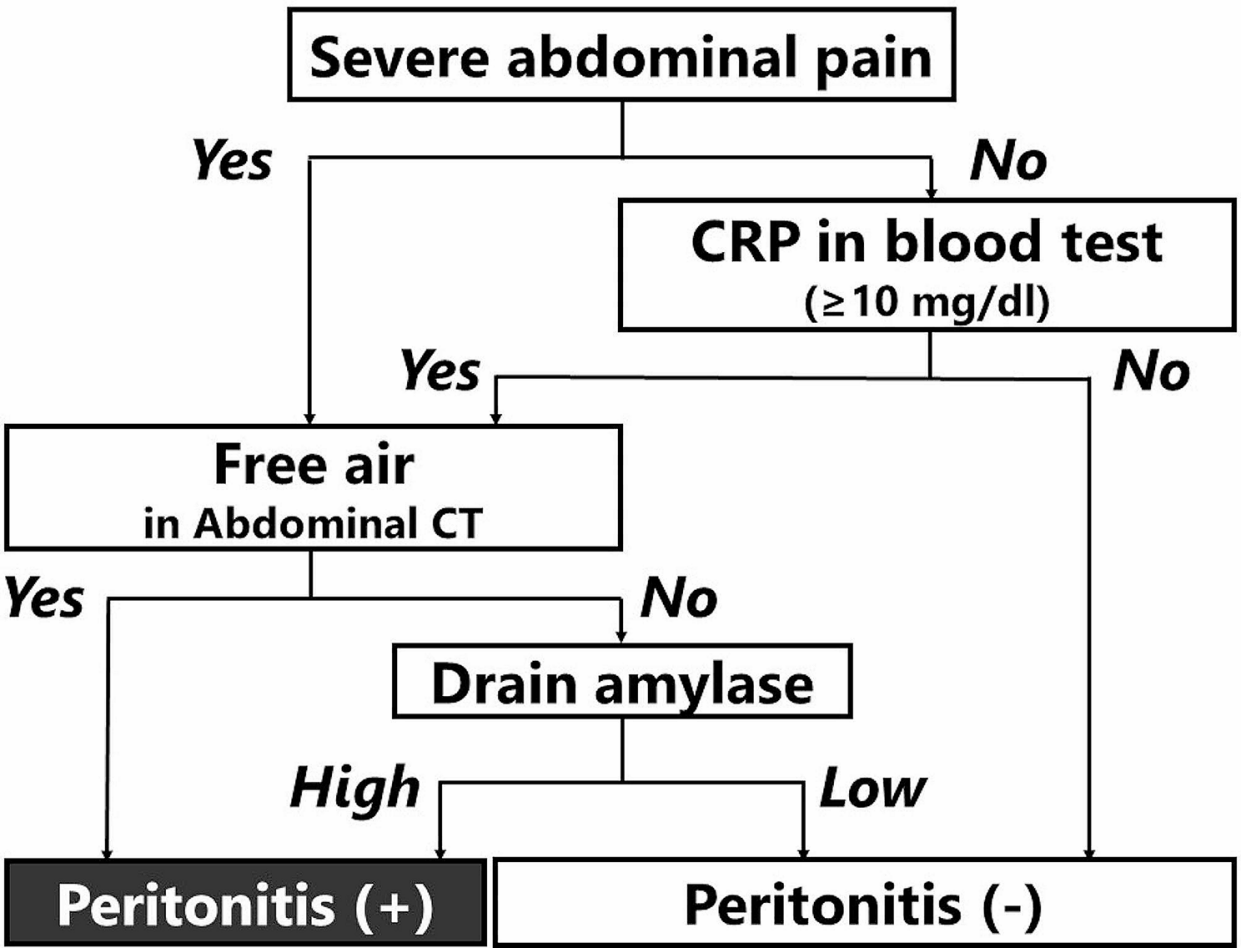
Flowchart for defining the occurrence of peritonitis after laparoscopic cell sheet transplantation. “Severe abdominal pain” means **1**) pain that could not be relieved with the application of a strong painkiller, such as pentazocine, or **2**) pain accompanied by rebound tenderness and muscle defense, as judged by two surgeons

Delayed perforation was defined as a perforation that manifested clinically, such as with a fever and abdominal pain. Fluid collection and abdominal air outside the duodenum on abdominal CT suggested perforation. We clarified the definition in detail to exclude the effects of laparoscopic procedures (Fig. 1).

- Safety outcome measures.

1. All adverse events during the follow-up period.
2. Any defect in the clinical trial product.
3. Any adverse event caused by a defect in the clinical trial product.

### Secondary endpoints

- Efficacy outcome measures.

1. Intraabdominal abscess during the follow-up period.
2. Postoperative drainage fluid examinations (amylase, bilirubin).
3. Development of epithelization or stricture on postoperative days 7 and 29.
4. Intraoperative procedural accidents.

- Perforation requiring intraoperative surgical closure.
- Intraoperative development of micro perforation.
- The onset of bleeding.
- Other procedural accidents.

5) Time spent on laparoscopic implantation of a myoblast sheet.
6) Success in the placement of a myoblast sheet.
7) Body temperature on the day after ESD and postoperative days 3 and 7.
8) Peripheral white blood cell counts on the day after ESD and on postoperative days 3 and 7.
9) CRP level on the day after ESD and postoperative days 3 and 7.
10) Presence or absence of abdominal pain during the follow-up period.
11) Presence or absence of the need for secondary emergency surgery owing to the onset of peritonitis after implantation of a skeletal muscle-derived cell sheet.
12) Presence or absence of bleeding requiring emergency care.
13) Curative resection rate in all tumors subjected to ESD.
14) Endoscopic mucosal resection size.
15) Evaluation of resected tumor specimen.

- Mucosal resection size.
- Histopathology data.

16) Relationship between success or failure of mucosal closure and the presence or absence of peritonitis after delayed perforation.

- Safety outcome measures.

1. Serious adverse events.
2. Adverse events caused by harvesting skeletal muscle tissues (adverse events with an undeniable causal relationship with harvesting skeletal muscle tissues).
3. Changes in vital signs, complete blood count, and serum chemistry.

### Autologous myoblast cell cultures and preparation of myoblast sheets

Seven weeks before duodenal ESD, approximately 2–5 g of skeletal muscle was obtained from the quadriceps muscle of each patient and transferred by air to the cell-processing faculty (CPF) of the Terumo Corporation (Kanagawa, Japan). In the CPF, the connective tissue will be carefully removed from the retrieved specimen, and the remaining muscle tissue was minced into small pieces. The muscles were digested at 37 °C in a aluminum block bath with TrypLE Select (Thermo Fisher, MA, USA) containing collagenase, gentamicin sulfate, and amphotericin B. The fluid was discarded, and culture medium (MCDB131; Thermo Fisher) supplemented with 20% fetal bovine serum was added to halt the enzymatic digestion process. Isolated cells were collected by centrifugation and then seeded onto flasks (Thermo Fisher) with MCDB131 medium (Thermo Fisher) supplemented with 20% fetal bovine serum.

After cultivation, they were harvested by trypsinization. After sufficient expansion for cell sheet fabrication, the cells were dissociated from the flasks with TrypLE Select, and the cell suspension will be cryopreserved and transferred to Nagasaki University Hospital two days before transplantation. In the CPF of Nagasaki University, the cells were reincubated on 60-mm temperature-responsive culture dishes (CellSeed, Tokyo, Japan) at 37 °C with the cell numbers adjusted to 2.2 × 10^7^ per dish. On the day of transplantation, the cells were washed with ice-cold HBSS(+) and incubated at room temperature for 10 min. After reducing the culture temperature, the myoblast sheet could be detached without any need for enzymatic treatment, thereby preserving the important membrane proteins and extracellular matrix and allowing the cell sheet to successfully integrate with the tissue at the implanted site. The diameter of each detached cell sheet was expected to be approximately 2.5 cm. To increase strength during handling, fibrin was sprayed onto the surface of the cell sheet.

### The evaluation of the myoblast sheet

The harvested myoblasts were assessed for the viable cell number and viability at every time point of passaging. Quality testing of the myoblast sheet also included assessments for the presence of bacteria, viruses, mycoplasma, or endotoxin contamination. Cell purity was measured by flow cytometry (Beckman Coulter, Miami, FL, USA) after staining with an anti-cluster of differentiation 56 antibody (CD56; BD Bioscience, San Diego, CA, USA).

## Results

The initial plan was to enroll six cases in our clinical study, but due to the coronavirus pandemic, which decreased the chance of detecting SNADETs through a medical examination with gastroduodenoscopy, this trial was limited to a total of three cases.

Table 3 summarizes the perioperative characteristics of the three enrolled patients. Laparoscopic transplantation of the two cell sheets was performed without any adverse events. Although the postoperative course was uneventful in all enrolled patients in this study, our strict criteria for the postoperative amylase level in drainage fluid determined that the development of peritonitis was positive in two of three cases.

**Table 3:** Clinical and operative characteristics and outcomes

	Case 1	Case 2	Case 3
Preoperative evaluation			
Tumor size (mm)	22	25	24
Tumor location	1st~2nd portion	2nd portion	2nd portion
		distal side of the papilla	distal side of the papilla
Total cell number	1.55 × 10^8^	1.80 × 10^8^	1.32 × 10^8^
CD56-positive cells (%)	95	98	98
Cellular viability (%)	98	97	95
ESD			
Size of resected tumor (mm)	34 × 25	41 × 22	37 × 25
Size of mucosal defect (mm)	43 × 31	43 × 30	51 × 40
Mucosal closure	complete	incomplete	complete
Intraoperative microperforation	no	no	yes, multiple
Number of transplanted sheets	2	2	2
Resected specimens			
en bloc resection	yes	yes	yes
Histology	adenoma	adenocarcinoma	adenocarcinoma
Vessel invasion	-	none	none
Postoperative course			
Peritonitis	no	yes	yes
Patient’s condition	stable	stable	stable
Hospital stay (days)	9	19	17
Duodenal stenosis after 7 weeks	no	no	no

### Case 1

**Fig. 2:**
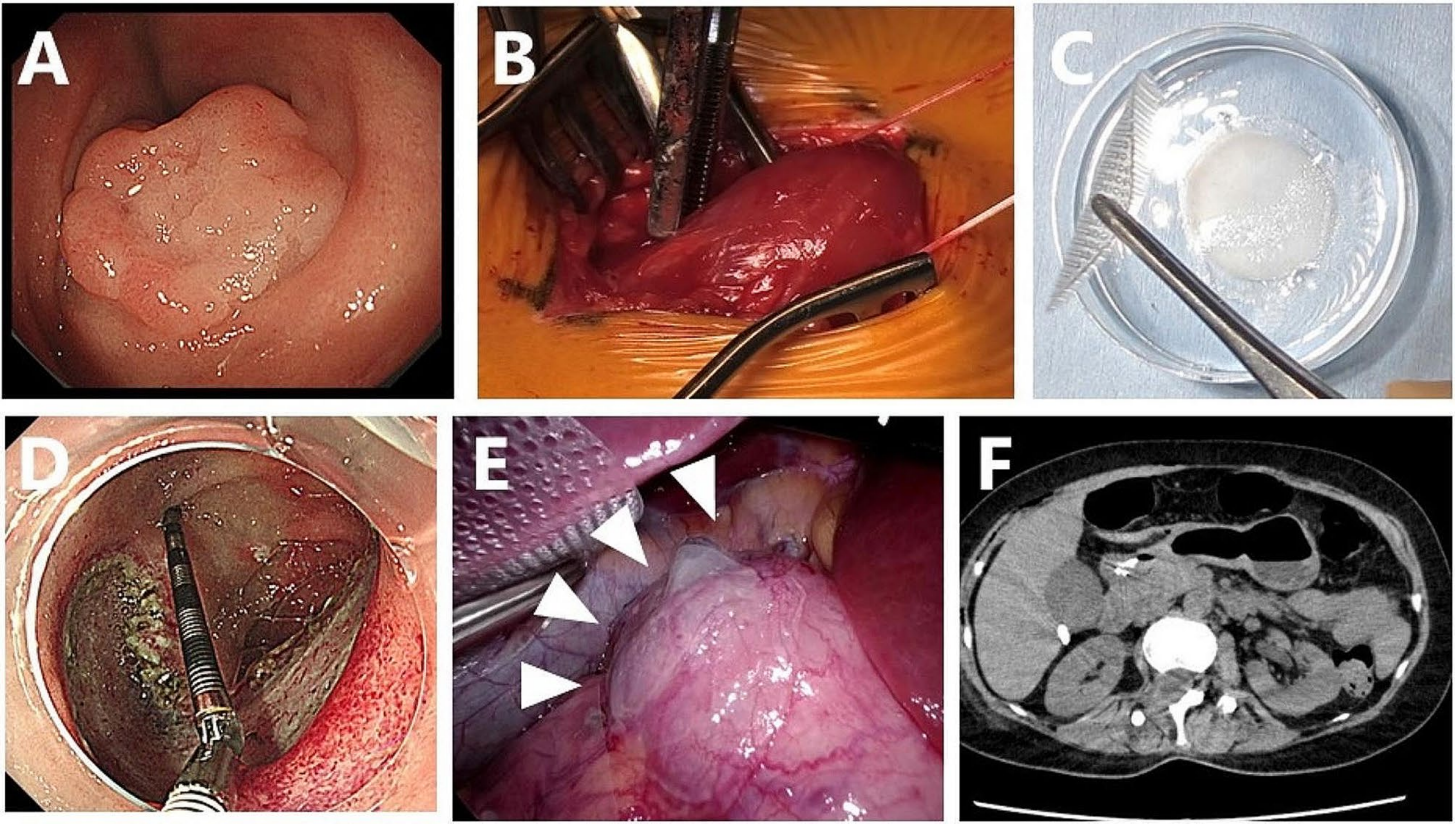
Preparation of the myoblast sheet and laparoscopic transplantation of the sheet after ESD in case 1. **A**: Superficial duodenal tumor located in the second portion of the duodenum. **B**: Muscle tissue was surgically excised from the femur of the patient. C: **A** transplantable myoblast sheet was harvested after seven weeks of culture. **D**: A mucosal defect around 40 mm in diameter after duodenal ESD. **E**: Cell sheets were transplanted onto the serosal side of the duodenal wall after ESD. **C**: Abdominal CT three days after transplantation revealed no obvious signs of perforation

Mid 50’s woman was referred to our hospital because SNADET was detected during a routine medical checkup. Upper gastroduodenal endoscopy revealed 0-IIa type tumors in the descending duodenum. As the tumor was 25 mm in diameter, endoscopic resection was indicated because of the potential for malignancy (Fig. 2A).

Written informed consent for inclusion in our clinical study was obtained, and muscle specimens were harvested from the vastus medialis muscle under local anesthesia (Fig. 2B). The resected specimen (3.2 g) was immediately transported by air under sterile conditions to the CPF of Terumo Corporation located at Kanagawa, where all culture and cell fabrication steps mentioned above were performed.

After seven weeks of cell expansion, the cell suspensions were transported back to the CPF of Nagasaki University Hospital on the day before transplantation and placed in temperature-responsive cell culture dishes (UPCell; CellSeed) to fabricate a myoblast sheet (Fig. 2C).

Under general anesthesia, duodenal LECS was performed: five trocars were initially inserted into the abdomen for laparoscopic transplantation of cell sheets. Intraoperative endoscopy revealed 0-IIa type tumors on the oral side of the papilla of Vater, in the descending duodenum. After clamping the jejunum, duodenal ESD was performed by an endoscopist (Fig. 2D). Although immediate perforation was not evident, the wall after ESD was so thin that the endoscopic light could be seen laparoscopically.

After closure of the mucosal defect with endoscopic clipping, two myoblast sheets were transplanted onto the severe side of the duodenal ESD site using a silicon-made membranous device. A myoblast sheet on the carrier was placed onto a polyester mesh, and the carrier and mesh were pinched with conventional laparoscopic forceps and rolled up for placement into the abdominal cavity through a 12-mm laparoscopic port. The carrier and nylon mesh were deployed, and the carrier attached to the cell sheet was placed on the surface of the duodenum, cell-sheet side down. After confirming the attachment of the cell sheet, the carrier and nylon mesh were gently removed and retrieved from the intra-abdominal cavity (Fig. 2E).

On postoperative day 1, the patient did not show any signs of peritonitis, such as abdominal pain or a fever. An endoscopic examination revealed no perforation, but dropout of several clips was observed. The amylase value in the fluid of the drain placed in Morrison’s pouch was 68 U/L on postoperative day 1.

Abdominal CT on postoperative day 3 showed neither air bubbles nor fluid around the duodenum (Fig. 2F), and there were no signs of peritonitis or retroperitonitis on a physical examination. The postoperative course was uneventful, and the patient was discharged from the hospital nine days after the operation. Follow-up examinations were performed approximately seven weeks after cell sheet transplantation, including endoscopic and clinical examinations. No tests revealed any abnormalities, confirming the safety of the transplantation procedure.

### Case 2

**Fig. 3:**
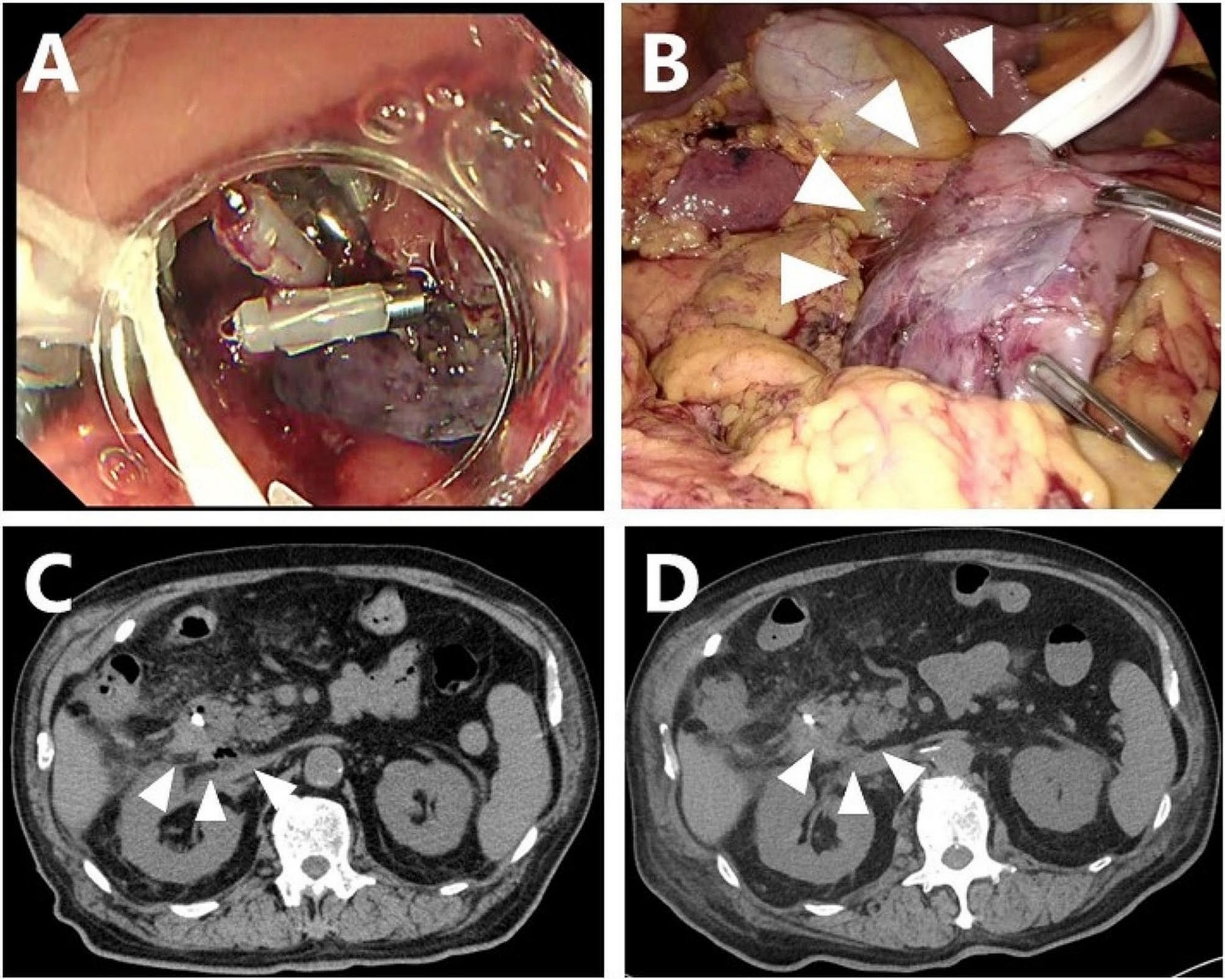
Myoblast sheet transplantation after ESD in case 2. **A**: The closure of the mucosal defect with clipping was incomplete due to its large diameter. A protruding, thinned ulcerative base after incomplete clipping was seen. **B**: Two cell sheets were transplanted to fully cover the thinned duodenal wall after duodenal mobilization. **C**: Abdominal CT three days after transplantation showed a small air bubble at the dorsal side of the duodenum. Neither massive free air nor fluid collection was evident. **D**: The air bubble on abdominal CT had diminished by seven days after transplantation

Mid 70’s man was referred to our hospital because a SNADET 25 mm in diameter was found in the second portion of the duodenum. Seven weeks after harvesting the skeletal muscle tissue, duodenal LECS was performed. Although no intraoperative perforation was observed, mucosal closure with endoscopic clips was insufficient because of the large mucosal defect after ESD (Fig. 3A). Incomplete closure of the mucosal defect by clipping causes the ulcer base to bulge outward, leaving a diverticulum-like space at the ESD site.

As the thinned area after ESD was semicircular from the dorsal side to the contralateral side of the pancreas, full mobilization of the duodenum was performed to the extent of the pancreatic head. Two cell sheets were applied to the protruding area of the ESD, and the omentum was placed onto the transplanted sheets (Fig. 3B).

Although there was no postoperative abdominal pain or a fever, an elevated intra-abdominal drain amylase level of 15,623 U/ml was observed the following day, and abdominal CT revealed a small amount of free air along the dorsal duodenum of the ESD site.

**Fig. 4:**
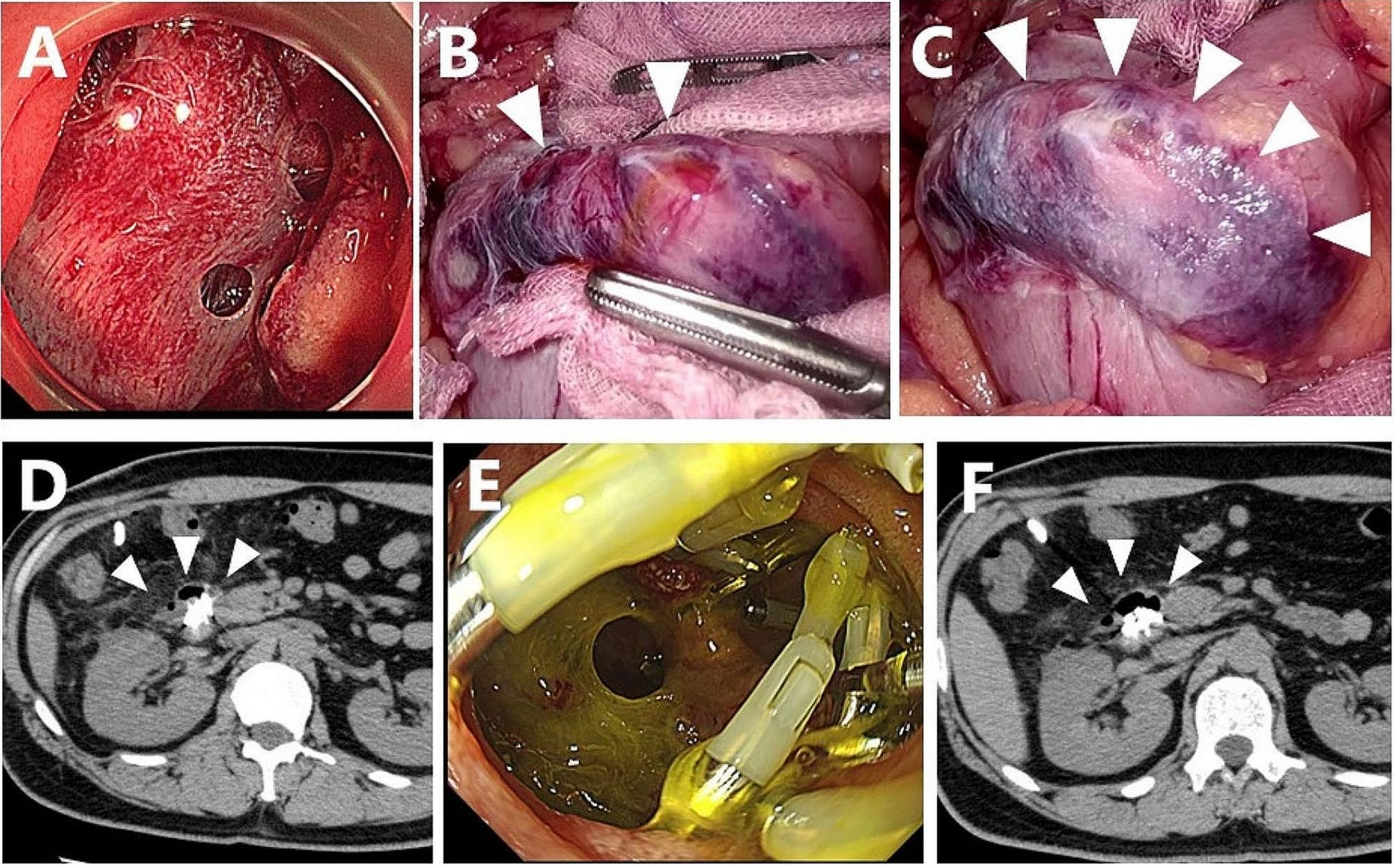
Postoperative changes in the excretion volume obtained from the drainage tube placed near the transplanted site in cases 2 (**A**) and 3 (**B**)

With close follow-up, the patient’s condition did not deteriorate, and the volume of drainage fluid and amylase value promptly decreased to 551 U/ml on the third day (Fig. 4A). Abdominal CT still showed free air bubbles on the dorsal side of the duodenum (Fig. 3C). There was no worsening of clinical symptoms, such as abdominal pain, and free air had completely diminished on abdominal CT (Fig. 3D).

On the 49th day after transplantation, endoscopic observation revealed no stenosis in the transplanted area.

### Case 3

**Fig. 5:**
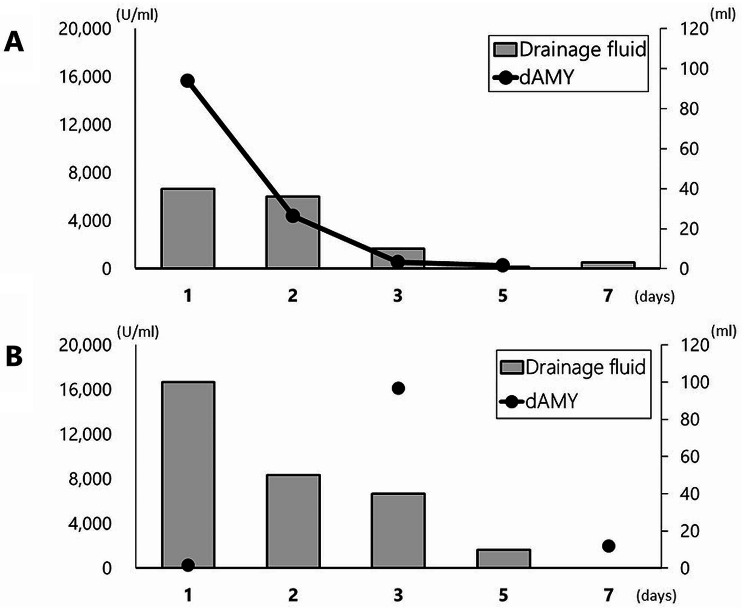
Myoblast sheet transplantation after ESD in case 3. **A**: At least four micro perforations were evident after duodenal ESD. **B**: Laparoscopy also revealed micro perforations with bile leakage. The thinned duodenal wall was protruding like the diverticulum due to mucosal clipping. **C**: All micro perforations were completely sealed by transplanted myoblast sheets. **D**: Abdominal CT three days after transplantation showed a small air bubble with diverticulum-like protrusion of the duodenal wall. Fluid collection around the duodenum was not evident. **E**: Endoscopy seven days after transplantation revealed an opening of the mucosa with dropout of intraoperative clipping. **F**: Abdominal CT seven days after transplantation showed air bubbles along the duodenal wall, but they had not extended even after endoscopic observation

Early 40’s man with a SNADET located in the descending portion of the duodenum was included in our study. During duodenal LECS, at least four micro perforations were observed intraoperatively; however, each perforation was small and not as severe as that accompanied by mucosal exfoliation (Fig. 5A). Air and bile leakage diminished after complete mucosal closure with endoscopic clips, where a thin ulcer base bulged outward like a diverticulum due to mucosal clips (Fig. 5B). Two cell sheets were applied to the serosa side after ESD to cover each perforated site, and the omentum was placed onto the sheets without fixation (Fig. 5C). No postoperative abdominal pain or a fever was observed; however, a blood test revealed a CRP level of 13 mg/dl.

The patient did not show any symptoms of peritonitis; however, an elevated drain amylase level of 16,109 U/ml was observed on postoperative day 3 (Fig. 4B). CT of the abdomen showed no marked increase in fluid collection and free air, but a small air bubble was detected on the lateral side of the ESD site (Fig. 5D). Two possibilities were considered as the source of this solitary air bubble: localized free air due to perforation or a diverticulum-like space in the duodenum. Since there were no worsening clinical symptoms, such as a fever or increased abdominal pain, protease inhibitors, somatostatin analog, and antibiotics were started for pancreatitis, resulting in a prompt reduction of the drain amylase level to 1,973 U/ml (Fig. 4B). On day 7, an endoscopic examination revealed an opening of the mucosa, which was closed intraoperatively with clips (Fig. 5E). A robust structure backing the bottom of the defect of the duodenal wall was observed, and abdominal CT after endoscopy showed no expansion of free air despite endoscopic insufflation (Fig. 5F).

On the 49th (50th) day after transplantation, endoscopic observation revealed no stenosis in the transplanted cell sheet area.

## Discussion

This clinical trial evaluated the efficacy, safety, and feasibility of laparoscopic transplantation of autologous myoblast sheets to prevent delayed perforation after duodenal ESD in patients with SNADETs. In this study, we showed that cell sheet transplantation was able to partially prevent postoperative perforation after duodenal ESD, even in patients with an increased risk of delayed perforation, such as those with incomplete mucosal closure and intraoperative micro perforations due to a larger tumor size than expected. The novelty lies in the fact that cell sheet transplantation was performed on the serosal side using a laparoscope and not on the inner lumen using an endoscope. The results obtained in this study are remarkable, as they can expand the applications of cell sheet medicine from the endoscopic field to the laparoscopic surgical field.

Recent advances in tissue engineering have enabled the use of cells as a promising modality for treating patients with intractable diseases. Cell therapy has recently been introduced into clinical practice for the functional repair of deficiencies in various fields, including hepatology and gastroenterology [24–31]. Cell sheet medicine has also been implemented in clinical practice in the field of cardiac surgery. Yoshikawa et al. reported that the advantages of cell sheet implantation include the potential to increase the number of implanted cells and the maintenance of cell-to-cell contact, which leads to a greater survival of cells, as well as a better paracrine effect than local injection [32, 33], which is thought to be the main mechanism involved in cell sheet transplantation. The secretion of various growth factors, such as hepatocyte growth factor (HGF) and vascular endothelial growth factor (VEGF), can locally promote angiogenesis at the transplanted site. We also recently indicated that myoblast sheet transplantation accelerates healing of the transplanted site through the chronological expression of various growth factors [34]. Based on the above findings, one of the mechanisms of cell sheet transplantation may therefore directly promote the regeneration of a thinned duodenal wall, and thereby strengthen the adhesion around the ESD site. In addition to these paracrine effects, the barrier effect of the myoblast sheet might also play a key role in the prevention of delayed perforation.

Cell sheet technology has also been applied in clinical practice in gastrointestinal fields as well. Ohki et al. conducted a clinical study of 10 patients in whom autologous buccal cell sheets were transplanted to cover mucosal defects after ESD for superficial esophageal cancer [35]. They demonstrated remarkable results in which cell sheet transplantation successfully prevented esophageal stricture, even after semi-circumferential ESD with a high risk of stenosis. In collaboration with Ohki et al., Yamaguchi et al. investigated the efficacy of cell sheet transplantation after airplane transportation of a fabricated cell sheet. Despite their study involving a wider resection area and lower cell sheet coverage for the ESD than those reported by Ohki et al., they also showed impressive results with a luminal stenosis rate of 40% and a median number of mandatory endoscopic dilatations of 0 [36]. Recently, the effect of cell sheet transplantation on post-anastomotic stenosis in congenital esophageal disease has also been reported. Among the three patients who received autologous oral mucosal cell sheets after endoscopic balloon dilatation for anastomotic stenosis, two were free from dilatation for at least one year after transplantation [37]. Regarding transplantation onto the esophageal lumen as described above, transplantation of the myoblast sheet directly into the mucosa under endoscopy seems to be an attractive option, however, there is still no available feasible device that allows us to transport a fragile cell sheet into the duodenum over the narrow esophagus, the esophagogastric junction and the pylorus. Moreover, the presence of bile, pancreatic juice and active peristalsis of the duodenal lumen is well known to make the engraftment of such a fragile cell sheet onto the ESD ulcer very difficult.

Various routes of cell transplantation, such as intravascular and local injection, are considered, depending on the site where the implanted cells are expected to function. Our study is important in that we successfully transported fragile myoblast sheets into a high-pressure pneumoperitonized abdominal cavity through a thin laparoscopic trocar. In endoscopic transplantation of cell sheets into the inner lumen of the esophagus, surgical intervention to create a specified route for transplantation is not required, and endoscopically applying a sheet-like structure is likely to be relatively easy with a simple procedure [37–39]. However, transplantation into the inner body, such as the thoracic and abdominal cavities, requires a surgical route for cell sheet transplantation.

In a clinical study of autologous myoblast sheet transplantation in 15 ischemic cardiomyopathy patients, the cell sheets were transplanted onto the left ventricular wall of the heart through thoracotomy of the left fifth intercostal space [40]. Kanzaki et al. reported thoracoscopic transplantation of an autologous dermal fibroblast sheet after lung resection to treat air leakage [41, 42]. They used a CellShifter with thoracoscopic equipment for transplantation through a trocar inserted into the thoracic wall. Recently, various thoracoscopic devices have been developed for the transplantation of sheets into bone-supported thoracic cavities. However, no reports have described the transplantation of sheets into a high-pressure pneumoperitonized abdominal cavity [43, 44]. We established a feasible procedure for laparoscopic transplantation of myoblast sheets in our porcine model [45] and confirmed that this procedure is also practical for clinical settings. This approach may expand the potential utility of cell sheet medicine to intra-abdominal organs other than the duodenum.

Despite inadequate endoscopic clip closure of a large mucosal defect (> 40 mm) in case 2 and multiple intraoperative micro perforations in case 3, cell sheet transplantation in both cases was able to prevent clinical problems, such as increased drainage or worsening of peritonitis symptoms due to rupture of the ulcer base. In case 2, we were unable to exclude the possibility that the elevation of drainage amylase might have been due to pancreatitis caused by Kocher’s maneuver extending to the dorsal side of the pancreas, rather than due to delayed perforation. It might also be possible that the micro perforations in case 3 were not completely covered by the cell sheet, as we stipulated that we could use only two cell sheets. As the patient’s postoperative condition did not deteriorate, mandatory reoperation for cure was not necessary. Although we should judge “peritonitis” occurred in two of three patients along with our criteria, our findings indicate the effectiveness of cell sheet transplantation for reinforcement of defects of the intestinal wall, even with exposure to irritant digestive juice.

An endoscopic examination revealed a robust structure backing the mucosal defect seven days after ESD in case 3. This finding was similar to those obtained in our animal model, indicating massive fibroblasts and collagen fibers among the implanted myoblast backing diminished ulcers based on an immunohistochemical examination at the ESD site [23]. In all cases, neither stenosis nor any recurrent tumor was found on endoscopy at 49 days after transplantation, which indicates that the paracrine effect of the cell sheet did not negatively impact the process of tissue regeneration.

With its recent national health insurance coverage, duodenal LECS accompanied by suture reinforcement of thin ESD sites has rapidly become popular. Proper candidates for cell sheet transplantation in the future may be patients in whom laparoscopic suture after ESD would be difficult due to the tumor being located on the pancreatic side and in cases of large lesions where there is concern about duodenal stricture. A randomized control trial is needed to validate the effectiveness of cell sheet transplantation for patients who truly benefit from this treatment.

However, we are concerned that there would be hurdles in the accumulation and registration of suitable patients for such a study. First, the subjects of that future study might be those with SNADETs without extension to the submucosa; however, the preoperative diagnosis of the depth of invasion with SNADETs as well as their histological diagnosis is tremendously difficult. In the present study, because tumors were judged as invading into deeper layers over the mucosa with preoperative endoscopy, three patients were excluded from enrollment before the eligibility committee (data not shown). However, a postoperative pathological examination after duodenal LECS revealed that one was an adenoma, and the other two were duodenal carcinomas confined to the mucosa. Second, a large discrepancy was found between the preoperative endoscopic tumor diameter measurement and the actual resected tumor diameter upon ESD, which might have led to the unpredictability of the difficulty of ESD and mucosal clipping after ESD. This uncertainty in the ESD procedure might interfere with the equal distribution of patients in randomized control trials.

There have been several reports on the application of cell sheet technology in the abdominal cavity. Maruya et al. reported that adipose-derived stem cell (ADSC) sheets enhanced anastomotic strength in a miniature pig model [46]. Hara et al. similarly reported the efficacy of ADSC sheet transplantation around the anastomotic site of the bile duct to prevent biliary anastomotic stricture [47]. In addition, we previously reported that cell sheets fabricated with islets, fibroblasts, or ADSCs had a cytoprotective effect on the islet function compared to sheets fabricated with islets alone [48]. These sheets may be transplanted onto the liver surface, as described by Inagaki et al. [49]. Miyamoto also reported that hepatocyte sheets transplanted onto the liver surface exerted sustained liver function stimulation compared with sheets transplanted subcutaneously [50].

Several limitations associated with the present study warrant mention. Because of the impact of the spread of the new coronavirus infection, this trial was limited to a total of only three cases, and because the procedure was performed for prevention, statistical consideration of efficacy is difficult. It was also not possible to rule out the influence of the small sample size on the study outcome and power calculations could therefore not performed to support the usefulness of this study..In addition, our clinical study was designed as a single-arm, single-center study that may have affected the outcome of therapy. A randomized control trial seemed difficult to perform, as described above, so future studies with more patients are needed.

## Conclusions

Our ongoing FIH clinical trial demonstrated successful transplantation of autologous myoblast sheets into pneumoperitonized abdominal cavities via the laparoscopic approach. These results suggest the potential application of cell sheet medicine in the treatment of various abdominal organs with minimal invasiveness. The efficacy of cell sheet transplantation for preventing delayed perforation after duodenal ESD must be further validated in subsequent clinical trials.

## Data Availability

The data that support the findings of this study are available from the corresponding author on reasonable request.
